# Quiz Compends. No. 1. Anatomy

**Published:** 1882-12

**Authors:** 


*Quiz·Compends No.* 1. *Anatomy. Questions on Human Anatomy.* By Saml. O. L. Potter, M. A., M. D., with sixty-three illustrations. Publishers: P. Blakiston, Son & Co., Philadelphia, 1882.

This work contains a series of questions and answers, comprising a concise description of the bones, articulations, muscles, arteries, veins, absorbents, and nerves of the human body, including the heart and brain as essential parts of the circulating and nervous systems respectively.

It is intended for the use of medical students, and must prove to be a valuable assistant in preparing for examinations. The text closely follows Gray, and the Latin names universally used by anatomists have been retained. Its small size, and flexible cover make it convenient for a pocket reference, and the style of its publication is commendable.

